# Interaction of methionine sulfoxide reductase B5 with SlMYC2 stimulates the transcription of MeJA-mediated autophagy-related genes in tomato fruit

**DOI:** 10.1093/hr/uhad012

**Published:** 2023-02-01

**Authors:** Dedong Min, Fujun Li, Maratab Ali, Jiong Liu, Xiaodong Fu, Yanan Song, Jun Ding, Xiaoan Li, Nana Ji, Xinhua Zhang

**Affiliations:** School of Agricultural Engineering and Food Science, Shandong University of Technology, Zibo 255000, Shandong, China; School of Agricultural Engineering and Food Science, Shandong University of Technology, Zibo 255000, Shandong, China; School of Agricultural Engineering and Food Science, Shandong University of Technology, Zibo 255000, Shandong, China; School of Food and Agricultural Sciences, University of Management and Technology, Lahore 54000, Pakistan; School of Agricultural Engineering and Food Science, Shandong University of Technology, Zibo 255000, Shandong, China; School of Agricultural Engineering and Food Science, Shandong University of Technology, Zibo 255000, Shandong, China; School of Agricultural Engineering and Food Science, Shandong University of Technology, Zibo 255000, Shandong, China; School of Agricultural Engineering and Food Science, Shandong University of Technology, Zibo 255000, Shandong, China; School of Agricultural Engineering and Food Science, Shandong University of Technology, Zibo 255000, Shandong, China; School of Agricultural Engineering and Food Science, Shandong University of Technology, Zibo 255000, Shandong, China; School of Agricultural Engineering and Food Science, Shandong University of Technology, Zibo 255000, Shandong, China

## Abstract

Methyl jasmonate (MeJA) has been shown to induce autophagy in various plant stress responses and metabolic pathways. MYC2 is involved in MeJA-mediated postharvest fruit biological metabolism, but it is unclear how it affects MeJA-induced fruit autophagy. In this study, we noticed that silencing *SlMYC2* significantly reduced the increase in *autophagy-related genes* (*SlATGs*) expression induced by MeJA. SlMYC2 could also bind to the promoters of several *SlATGs*, including *SlATG13a*, *SlATG13b*, *SlATG18a*, and *SlATG18h*, and activate their transcript levels. Moreover, SlMsrB5, a methionine sulfoxide reductase, could interact with SlMYC2. Methionine oxidation in SlMYC2 and mimicking sulfoxidation in SlMYC2 by mutation of methionine-542 to glutamine reduced the DNA-binding ability and transcriptional activity of SlMYC2, respectively. SlMsrB5 partially repaired oxidized SlMYC2 and restored its DNA-binding ability. On the other hand, silencing *SlMsrB5* inhibited the transcript levels of *SlMYC2*-targeted genes (*SlATG13a*, *SlATG13b*, *SlATG18a,* and *SlATG18h*). Similarly, dual-luciferase reporter (DLR) analysis revealed that SlMsrB5–SlMYC2 interaction significantly increased the ability of SlMYC2-mediated transcriptional activation of *SlATG13a*, *SlATG13b*, *SlATG18a,* and *SlATG18h*. These findings demonstrate that SlMsrB5-mediated cyclic oxidation/reduction of methionine in SlMYC2 influences *SlATGs* expression. Collectively, these findings reveal the mechanism of SlMYC2 in *SlATGs* transcriptional regulation, providing insight into the mechanism of MeJA-mediated postharvest fruit quality regulation.

## Introduction

Autophagy is an emerging cellular process that degrades cytoplasmic components to maintain cellular homeostasis [1, 2]. Studies have found that autophagy is required for plant development and stress responses [3]. Autophagy is also important in regulating postharvest fruit and vegetable quality to reduce postharvest losses [4–6]. During the autophagy process, the formation of autophagosomes is essential for increased autophagic activity, which is regulated by autophagy-related genes (*ATGs*) [7]. Inducing *ATGs* may thus be important for postharvest fruit quality by regulating autophagic activity.

Methyl jasmonate (MeJA) is a signaling molecule that regulates various physiological metabolisms in postharvest fruits, such as ripening, senescence, secondary metabolism, and stress responses [8, 9]. Moreover, MeJA was found to have an induction effect on *ATGs* in postharvest Chinese flowering cabbage [5] and tomato fruit [6], revealing the underlying mechanism of MeJA in postharvest fruit and vegetable quality retention. Because MeJA-regulated *ATGs* expression has been shown to correlate with postharvest fruit quality control, it would be beneficial to explore the potential molecular mechanism by which MeJA promotes *ATGs* expression in postharvest fruit.

MYC2 is the master regulator of the jasmonic acid (JA) signaling pathway, which regulates secondary metabolism and stress responses in plants [10–12]. Several studies have demonstrated that the transcription factor MYC2 can regulate secondary metabolism by binding to the promoters of target genes. For example, MdMYC2 enhanced ethylene production during ripening in apple by binding specifically to the promoters of ethylene biosynthesis genes [13]. Moreover, SlMYC2 could be involved in MeJA-mediated polyamine biosynthesis by specifically binding and activating *arginase genes SlARG1* and *SlARG2*, *arginine decarboxylase gene SlADC,* and *ornithine decarboxylase gene SlODC*, all of which contribute to postharvest tomato fruit chilling tolerance [14]. Meanwhile, SlMYC2 also promotes MeJA-induced postharvest tomato fruit disease resistance [15]. These studies reveal that MYC2 is essential for MeJA-regulated postharvest fruit metabolism. However, the mechanism by which MYC2 regulates transcription levels of *ATGs* is not yet well understood.

**Figure 1:**
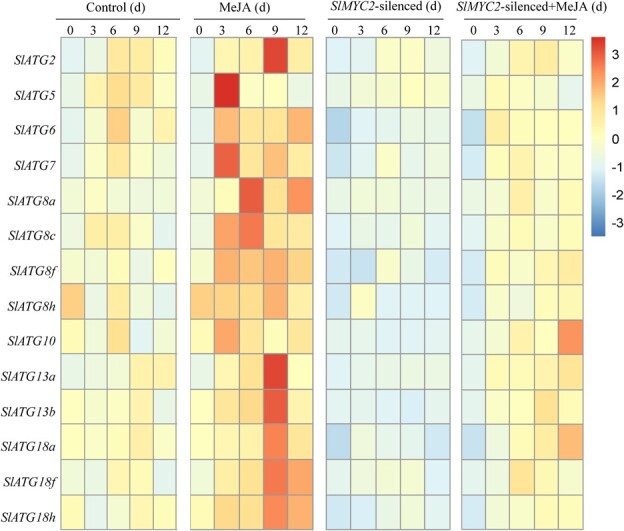
Effect of *SlMYC2* on MeJA-mediated expression of *SlATGs* in postharvest tomato fruit. The data were normalized based on the expression levels of *SlATGs.* Red and blue colors, respectively, indicate high and low abundance (color key scale right of the heatmap).

Although transcription factors can directly bind to the promoters of genes to activate or inhibit gene transcript levels, post-translational modifications of transcription factors are also critical in stress responses [16, 17]. Under stress conditions, reactive oxygen species (ROS) can over-accumulate and result in oxidative damage to macromolecules such as DNA, proteins, polysaccharides, and lipids, which eventually causes deterioration of postharvest fruit quality [18, 19]. In addition, proteins are the major targets of ROS, which cause the oxidation of the peptide backbone and amino acid side chains. Methionine (Met), one of the amino acids in protein, is particularly susceptible to ROS [20]. In the case of proteins, the oxidation of Met that can adversely affect protein function is reversible, which can be reduced by methionine sulfoxide reductase (Msr) [19, 20]. In our preliminary study, we screened SlMsrB5, a potential interacting protein of SlMYC2. SlMsrB5 belongs to the MsrB family, which could catalyse the reduction of methionine sulfoxide (MetSO) [21, 22]. Previous research has demonstrated the critical role of MsrB in various stress responses, including pathogen invasion in pepper [23] and drought in tomato [24]. Moreover, recent studies have suggested that sulfoxidation modification of the Msr-mediated transcription factor is a typical post-translational modification that can regulate various biological metabolisms by modifying protein function [21]. Tomato fruit ripening was influenced by the MsrB2-mediated redox modification of NOR [25], and banana ripening was associated with the MaMsrB2-mediated redox modification of MaNAC42 [26]. Little research has been done on the relationship between SlMYC2, SlMsrB5, and *SlATGs* transcription regulation.

Thus, this study investigated the role of SlMYC2 in regulating *SlATGs* transcripts using *SlMYC2*-silenced tomato fruit and analysed the targeted genes of SlMYC2 in regulating autophagy using an electrophoretic mobility shift assay (EMSA) and the dual-luciferase reporter (DLR). Furthermore, the interaction between SlMYC2 and SlMsrB5 was validated, and the role of this interaction was revealed in autophagy regulation in postharvest tomato fruit.

## Results

### SlMYC2 positively regulated the transcript levels of *SlATGs*

To explore the role and mechanism of SlMYC2 in MeJA-mediated postharvest fruit autophagic activity, the *SlMYC2*-silenced tomato fruit was constructed by virus-induced gene silencing (VIGS) and validated by quantitative real-time PCR (qRT-PCR). The result indicated that *SlMYC2* was lower in *SlMYC2*-silenced tomato fruit than in the control fruit as validated in our previous research [15], indicating that *SlMYC2*-silenced fruit was suitable for further analysis.

Then, we found that MeJA treatment could induce *SlATGs* expression compared to the control fruit (Fig. 1; Table S1, see online supplementary material). However, silencing *SlMYC2* significantly reduced the induction effect of MeJA treatment on *SlATGs* expression levels. *SlATGs* expression levels were lowest in *SlMYC2*-silenced fruit compared to other treatments.

### The transcription factor SlMYC2 specifically bound to the promoters of *SlATGs* and activated their expression

**Figure 2:**
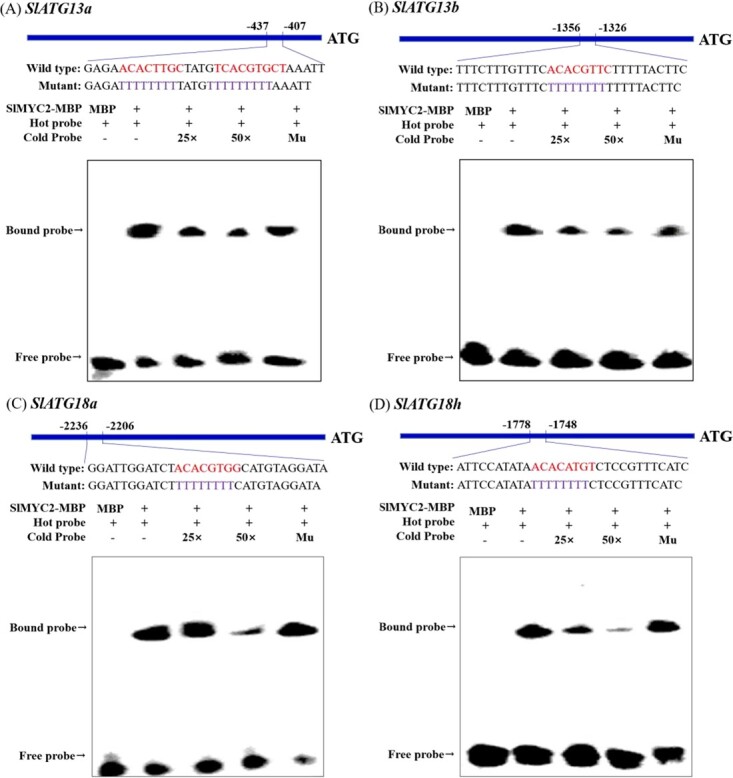
EMSA identified the binding of the transcription factor SlMYC2 to the promoters of *SlATG13a* (**A**), *SlATG13b* (**B**), *SlATG18a* (**C**)*,* and *SlATG18h* (**D**). Each probe was shown, with red letters representing the G/E-box and purple letters representing the mutant G/E-box (the sequence of G/E-box was replaced with TTTTTTTT). The cold probe that was unlabeled with biotin was regarded as competition. ‘+’ represented addition, while ‘−‘ represented no addition.

It has been reported that transcription factor MYC2 can recognize the G/E-box elements in the promoters of target genes [27]. Promoter analysis indicated that MYC2 had a higher binding ability with the promoter sequences of *SlATG13a*, *SlATG13b*, *SlATG18a*, and *SlATG18h.* Thus, the probes containing MYC2-binding site (G/E-box elements) were designed, which were used for EMSA to investigate whether SlMYC2 could bind to the promoters of four selected genes. The results showed that the SlMYC2-MBP (MBP, maltose binding protein) fusion protein could specifically bind to the probes of *SlATG13a*, *SlATG13b*, *SlATG18a*, and *SlATG18h*, causing mobility shift (Fig. 2). Next, competition-binding experiments were carried out using the cold probes, indicating the intensity of mobility shift was reduced by increasing the amount of cold probe. To further confirm the specific binding site of SlMYC2, the mutant probe (sequence of G/E-box in the cold probe was replaced with TTTTTTTT) was synthesized. However, the addition of the mutation probe did not affect the binding ability of SlMYC2 to *SlATG13a*, *SlATG13b*, *SlATG18a,* and *SlATG18h* promoters, indicating SlMYC2 could directly target the promoters of *SlATG13a*, *SlATG13b*, *SlATG18a,* and *SlATG18h* by binding to the G/E-box.

**Figure 3:**
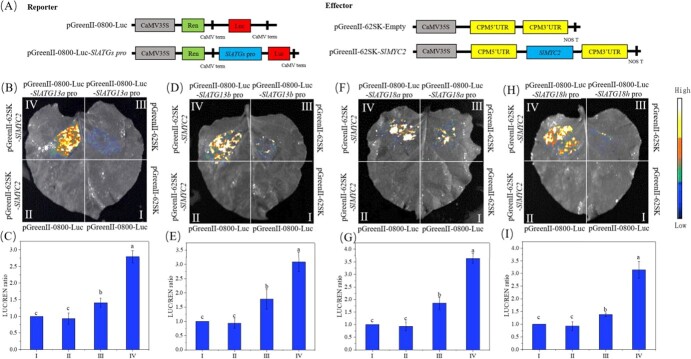
The dual-luciferase reporter assay verified the regulatory effects of the transcription factor SlMYC2 on the transcription of *SlATG13a, SlATG13b*, *SlATG18a,* and *SlATG18h*. (**A**) Schematic diagram of the reporter and effector vectors. **B**, **D**, **F**, and **H** Luciferase imaging assay of tobacco leaves. **C**, **E**, **G**, and **I** LUC/REN ratio. Region I represented the part of the tobacco leaf that was co-infected with pGreenII 0800-LUC and pGreenII-62SK; Region II represented the part of the tobacco leaf that was co-infected with pGreenII-0800-LUC and pGreenII-62SK-*SlMYC2*; Region III represented the part of the tobacco leaf that was co-infected with pGreenII-0800-LUC-pro and pGreenII-62SK; Region IV represented the part of the tobacco leaf that was co-infected with pGreenII-0800-LUC-pro and pGreenII-62SK-*SlMYC2*. Different letters represent significant differences (*P* < 0.05).

Furthermore, the regulatory effects of the transcription factor SlMYC2 on *SlATG13a*, *SlATG13b*, *SlATG18a,* and *SlATG18h* were assayed using the DLR. SlMYC2 driven by the CaMV 35S promoter was employed as an effector. The LUC gene driven by each promoter of *SlATG13a*, *SlATG13b*, *SlATG18a,* and *SlATG18h,* and the REN gene driven by the CaMV 35S promoter were employed as reporters and internal control, respectively (Fig. 3A). The highest luminescence intensity was detected in region IV, which was co-infected with pGreenII-62SK-*SlMYC2* and pGreenII-0800-Luc-*SlATGs* pro (Fig. 3B, D, F, and H). A weak luminescence intensity was detected in leaves co-infected with pGreenII-62SK and pGreenII-0800-Luc-*SlATGs* pro (Region III). Furthermore, no luminescence signal was detected in leaves infected with pGreenII-62SK + pGreenII-0800-Luc (regions I) nor pGreenII-62SK-*SlMYC2* + pGreenII-0800-Luc (regions II). Consequently, the ratio of firefly luciferase to renilla luciferase (LUC/REN) in region IV was significantly higher than in other regions (Fig. 3C, E, G, and I). Our findings confirmed that the transcription factor SlMYC2 could significantly activate the transcript levels of *SlATG13a*, *SlATG13b*, *SlATG18a,* and *SlATG18h*.

### Interaction of SlMYC2 with SlMsrB5

To investigate the potential molecular mechanism involved in MeJA-SlMYC2 mediated transcriptional regulation of *SlATGs*, the coding sequence (CDS) of *SlMYC2* was cloned into pGBKT7 vector as bait vector pGBKT7-*SlMYC2* to screen its interacting proteins in the tomato cDNA library. Among the 82 putative interacting proteins after the screening, a positive colony named SlMsrB5 (Uniprot: A0A3Q7IFG3) was further analysed because it has been reported that SlMsrB5 was critical in postharvest fruit disease defense [28].

**Figure 4:**
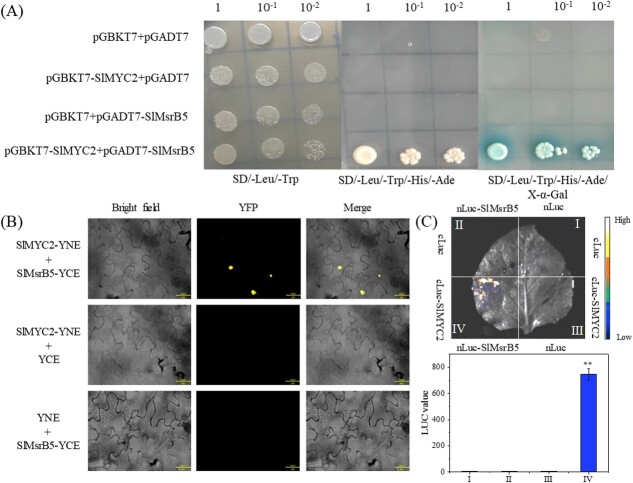
Verification of the interaction of SlMYC2 and SlMsrB5. **A** Y2H assay. Yeast cells grew on SD/−Trp/−Leu, SD/−Trp/−Leu/-His/−Ade, and SD/−Trp/−Leu/-His/−Ade/X-α-Gal at 29°C each. **B** BiFC assay. The CDS (coding sequences) of *SlMYC2* and *SlMsrB5* were inserted into the C- and N-terminus of YFP, respectively, and then co-injected into tobacco leaves. The fluorescence signal of YFP was detected. **C** LCI assay. The different combinations (cLuc+nLuc; cLuc+nLuc-SlMsrB5; cLuc-SlMYC2 + nLuc; cLuc-SlMYC2 + nLuc-SlMsrB5) comprising equal volumes of each vector were co-injected into tobacco leaves by the *Agrobacterium* strain GV3101. The LUC image and LUC value were determined.

According to the findings, SlMsrB5 was identified as one of the SlMYC2-interacting proteins. Next, the yeast two-hybrid (Y2H) assay was used to confirm the interaction between SlMYC2 and SlMsrB5, as shown in Fig. 4A. All yeast cells could survive on a synthetic medium lacking leucine and tryptophan (SD/-Leu/-Trp), but only yeast cells containing pGBKT7-*SlMYC2* and pGADT7-*SlMsrB5* grew on a synthetic medium lacking leucine, tryptophan, histidine, and adenine (SD/-Leu/-Trp/-His/-Ade), and turned blue in the presence of X-α-Gal, indicating that SlMYC2 and SlMsrB5 could physically interact in yeast.

Then, the interaction of SlMYC2 and SlMsrB5 was validated using the bimolecular fluorescence complementation (BiFC) assay (Fig. 4B). Our results indicated that nuclear localization signals of yellow fluorescent protein (YFP) were detected in tobacco leaves that were co-transfected with SlMYC2-YNE and SlMsrB5-YCE. However, no signals were detected in tobacco leaves that were co-transfected with SlMYC2-YNE + YCE nor YNE + SlMsrB5-YCE. In addition, the prediction of subcellular localization of SlMYC2 and SlMsrB5 was conducted using programs of ProtCompV.9.0 Server, Target P 2.0 Server and Cell-PLoc 2.0, indicating that SlMYC2 could co-localize with SlMsrB5 in the nucleus, which might explain why the fluorescence emitted by SlMYC2 interacting with SlMsrB5 appeared in the nucleus.

Moreover, the interaction of SlMYC2 and SlMsrB5 was also validated in tobacco using a firefly luciferase complementation imaging (LCI) assay. SlMsrB5 was inserted into the N-terminal region of the firefly luciferase (nLuc), whereas SlMYC2 was linked to the C-terminal region of the firefly luciferase (cLuc). Tobacco leaves co-infected with cLuc-SlMYC2 + nLuc-SlMsrB5 showed a high luminescence intensity and luciferase activity (Fig. 4C). Negative controls, such as nLuc-SlMsrB5/cLuc, nLuc/cLuc-SlMYC2, and nLuc/cLuc, revealed no luminescence signal. These results suggested that SlMYC2 and SlMsrB5 could interact in tobacco.

### Characteristics of SlMsrB5

**Figure 5:**
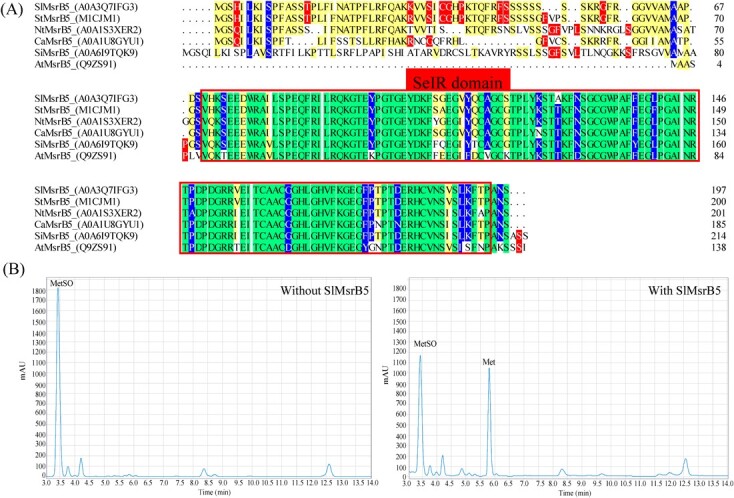
Characterization assay of SlMsrB5. **A** Multiple sequence alignment of SlMsrB5 with MsrB5 proteins from different species. The SeIR domain (76–194 amino acids) is shown in the red box. **B** HPLC analysis of the role of SlMsrB5 in catalysing MetSO reduction following treatment with control or SlMsrB5, indicating a decrease in MetSO and the production of Met in the presence of SlMsrB5. The peak corresponding to MetSO and Met was detected at 3.4 and 5.8 min, respectively.

Amino acid sequence analysis revealed that the sequence of SlMsrB5 comprised 197 amino acids with a predicted SeIR domain (one of the domains of MsrB) in the C-terminal. Multiple sequence alignment indicated that SeIR domain, containing 119 amino acids from 76 to 194 amino acids, in SlMsrB5 was highly conserved (Fig. 5A). Moreover, the ability of SlMsrB4 to reduce MetSO was investigated using high-performance liquid chromatography (HPLC). The result revealed that the addition of SlMsrB5 protein resulted in the production of Met and a decrease of MetSO (Fig. 5B).

### SlMsrB5 regulated the redox status of SlMYC2

**Figure 6:**
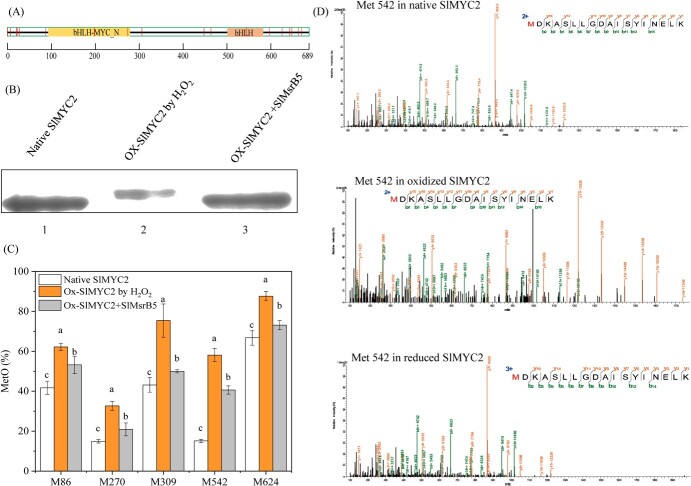
SlMsrB5 regulated the redox state of SlMYC2. **A** Simplified schematic diagram of SlMYC2. The red vertical bar represents the distribution of methionine in SlMYC2. **B** Electrophoretic mobility of SlMYC2 with different redox states as determined by SDS-PAGE. **C** The changes in the percentage of MetO in SlMYC2 with different redox states were determined by LC–MS/MS. Different letters represent significant differences (*P* < 0.05). **D** Mass spectrometry of peptides containing oxidized or reduced Met 542 in SlMYC2.

The SlMYC2 protein contains N-terminal bHLH-MYC domain (SlMYC2^94–277^) and C-terminal bHLH domain (SlMYC2^506–586^) (Fig. 6A). Then, the gel shift assay was performed to evaluate the influence of SlMsrB5 on the redox status of SlMYC2. Purified His-SlMYC2 was prepared and then oxidized by 1 mmol L^−1^ hydrogen dioxide (H_2_O_2_) after which the oxidized SlMYC2 was treated with DTT in the presence or absence of recombinant protein pCold-TF-SlMsrB5. The results showed that H_2_O_2_-oxidized SlMYC2 shifted upwards, whereas the addition of recombinant protein pCold-TF-SlMsrB5 restored the migration pattern similar to oxidized SlMYC2 (Fig. 6B).

Afterward, the peptides from native SlMYC2, oxidized SlMYC2, and SlMsrB5-repaired oxidized SlMYC2 were analysed using liquid chromatography–tandem mass spectrometry (LC–MS/MS). The results indicated that there was more oxidized Met in peptide fragments containing Met542 in oxidized SlMYC2 than in native SlMYC2. Furthermore, the addition of recombinant protein pCold-TF-SlMsrB reduced the amount of oxidized Met (Fig. 6C). The detailed mass data of the peptide with different redox statuses were shown in Fig. 6D. These findings suggested that SlMsrB5 was a significant reducer of oxidized SlMYC2 protein.

### Sulfoxidation or mimicking sulfoxidation in SlMYC2 decreases its DNA-binding and transcriptional activity

**Figure 7:**
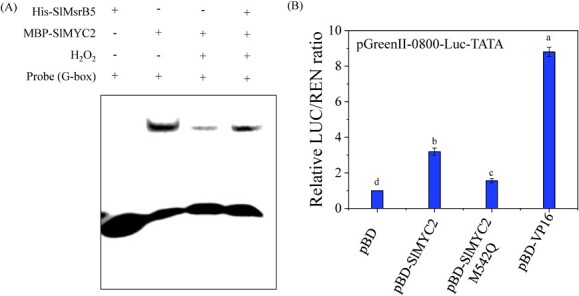
Methionine sulfoxidation decreased the DNA-binding activity and transcription activity of SlMYC2. **A** The EMSA indicated that methionine sulfoxidation in SlMYC2 reduced its DNA binding activity, which could be restored by the addition of SlMsrB5. **B** Mimicking Met-542 sulfoxidation inhibited the transcription activity of SlMYC2 *in vivo*. The pBD and pBD-VP16 vectors were used as the negative and positive control, respectively. Different letters represent significant differences (*P* < 0.05).

The DNA-binding ability of SlMYC2, oxidized SlMYC2, and SlMsrB5-repaired SlMYC2 was determined using the EMSA *in vitro*. The results indicated that SlMYC2 oxidation by H_2_O_2_ inhibited the binding ability of SlMYC2 to the G-box motif, which was restored by the addition of recombinant protein pCold-TF-SlMsrB (Fig. 7A).

The SlMYC2 protein had 18 Met residues, one of which was located in the bHLH domain (Fig. 6A) which was critical for its DNA-binding ability [11]. Thus, we hypothesized that Met542 sulfoxidation might be important in regulating SlMYC2 function. To validate the hypothesis, Met542 in SlMYC2 was mutated to glutamine (Q), which mimicked Met sulfoxidation (Fig. S1, see online supplementary material). Also, the effect of mimicking Met542 sulfoxidation on SlMYC2 transcriptional activity was investigated. The pBD and pBD-VP16 vectors were used as the negative and positive control, respectively. The results showed that both pBD-SlMYC2 and transcriptional activator control pBD-VP16 largely enhanced LUC/REN ratio in comparison with pBD-empty vector control. However, mimicking Met542 sulfoxidation in SlMYC2 (pBD-SlMYC2 M542Q) significantly reduced the LUC/REN ratio more than pBD-SlMYC2, suggesting that mimicking Met542 sulfoxidation reduced SlMYC2 transcriptional activity (Fig. 7B).

### *SlMsrB5* positively regulated MeJA–SlMYC2-mediated *SlATGs* transcription

**Figure 8:**
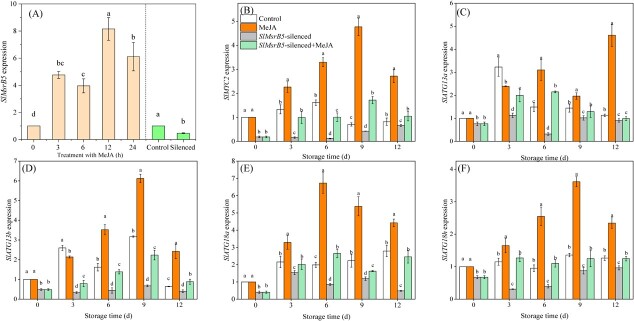
The role of SlMsrB5 on MeJA–SlMYC2-mediated *SlATGs* transcription. **A** Relative expression levels of *SlMsrB5* responding to MeJA treatment and in *SlMsrB5*-silenced tomato fruit. The effect of *SlMsrB5* silencing on transcript levels of *SlMYC2* (**B**), *SlATG13a* (**C**), *SlATG13b* (**D**), *SlATG18a* (**E**), and *SlATG18h* (**F**) during storage time. Different letters represent significant differences (*P* < 0.05).

As shown in Fig. 8A, MeJA treatment strongly activated *SlMsrB5* expression, which peaked at 12 h. To further analyse the role of SlMsrB5 in MeJA–SlMYC2-mediated *SlATGs* transcription, the *SlMsrB5*-silenced fruit was constructed (Fig. 8A). Silencing *SlMsrB5* largely inhibited the expression of *SlMYC2* and attenuated the induction of MeJA on *SlMYC2* transcription (Fig. 8B). Furthermore, silencing *SlMsrB5* reduced the expression levels of SlMYC2-targeted genes, including *SlATG13a*, *SlATG13b*, *SlATG18a,* and *SlATG18h*, which were partially restored by MeJA treatment (Fig. 8C–F).

**Figure 9:**
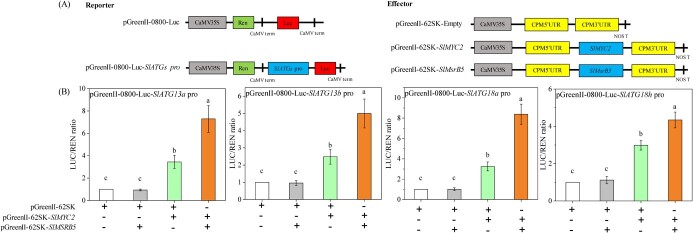
The transcription factor SlMYC2 interacted with SlMsrB5 to regulate *SlATG13a*, *SlATG13b*, *SlATG18a,* and *SlATG18h*. (**A**) Schematic diagram of the reporter and effector vectors. (**B**) The LUC/REN ratio assay verified the effect of SlMsrB5–SlMYC2 interaction on SlMYC2-regulated expression of *SlATG13a*, *SlATG13b*, *SlATG18a,* and *SlATG18h.* The effector and reporter vectors were co-injected into tobacco leaves. The values of LUC and REN were determined after 3 days. The symbols ‘−’ and ‘+’ represent absence and presence, respectively. Different letters represent significant differences (*P* < 0.05).

The DLR was performed to further investigate the influence of SlMsrB5 and SlMYC2 interaction on SlMYC2-mediated *SlATGs* expression (Fig. 9). In agreement with the preceding findings, the LUC/REN ratios in tobacco leaves co-infected with pGreenII-62SK-*SlMYC2* and pGreenII-0800-Luc-*SlATGs* pro were higher than those in tobacco leaves co-infected with pGreenII-62SK and pGreenII-0800-Luc-*SlATGs* pro. Furthermore, the highest LUC/REN ratios were found in tobacco leaves co-infected with pGreenII-62SK-*SlMYC2*, pGreenII-62SK-SlMsrB5, and pGreenII-0800-Luc-*SlATGs* pro, indicating that the interaction between SlMYC2 and SlMsrB5 enhanced the activation effects of SlMYC2 on *SlATG13a*, *SlATG13b*, *SlATG18a,* and *SlATG18h*.

## Discussion

### SlMYC2 was involved in the regulation of *SlATGs* expression levels

Autophagy is an important regulatory pathway involved in nutrient remobilization during plant development, senescence, and stress responses [29, 30]. Previous studies have indicated that autophagy is also involved in postharvest fruit quality regulation [4–7]. Therefore, increasing *SlATGs* transcript levels may play a significant role in a variety of physiological metabolic processes in postharvest fruit.

MeJA, as an important plant hormone, plays a critical role in maintaining the postharvest quality of fruit by interacting with plant hormone biosynthesis and transduction, regulating enzyme activities, and promoting secondary metabolism [9, 31, 32]. Moreover, the induction effect of MeJA on autophagic activity was noticed in postharvest Chinese flowering cabbage [5] and tomato fruit [6], implying that MeJA may maintain postharvest fruit quality by regulating autophagic activity. In this study, MeJA treatment significantly induced *SlATGs* transcript levels in tomato fruit during storage, a result consistent with reported findings [5, 6]. Thus, MeJA may regulate postharvest fruit quality by activating *ATGs* expression and inducing autophagy. However, the molecular mechanism by which MeJA affects *SlATGs* transcription is still unknown.

MYC2 functions as a key regulator in the JA pathway, allowing it to play an important role in MeJA-mediated plant metabolism [10–12]. For example, SlMYC2 was found to be involved in MeJA-triggered tomato leaf senescence by directly regulating a chlorophyll degradation enzyme-encoding gene, *SlPAO*, and photosynthesis-related genes, *SlRCA* and *SlSBPASE* [33]. Moreover, SlMYC2 was also found to be involved in postharvest tomato fruit resistance to cold stress [34] and *Botrytis cinerea* [15]. In this study, we found that silencing *SlMYC2* significantly inhibited the enhancement of *SlATGs* expression levels induced by MeJA, thus showing that SlMYC2 might positively regulate MeJA-mediated *SlATGs* expression.

The transcription factor MYC2 participates in various biological processes in plants by binding to target gene promoters. For example, MYC2 positively regulated abscisic acid biosynthesis in *Arabidopsis* by directly binding to the *ABA2* promoter [35]. In addition, MYC2 was found to be involved in proline synthesis via binding to the promoter of *pyrroline-5-carboxylate synthase 1* (*P5CS1*) to regulate salt stress [27]. Based on such findings, it is worthwhile to investigate whether SlMYC2 specifically regulates *SlATGs*, which will provide evidence for the molecular mechanism of MeJA-mediated transcriptional regulation of *SlATGs*. As a result of the EMSA and DLR assays, SlMYC2 bound directly to the promoters of *SlATG13a*, *SlATG13b*, *SlATG18a,* and *SlATG18h,* and activated their expression. Thus, SlMYC2 might be involved in MeJA-mediated postharvest fruit quality regulation by specifically activating *SlATGs* expression.

### SlMsrB5 participated in MeJA–SlMYC2-mediated transcriptional regulation of *SlATGs* by interacting with SlMYC2

Methionine oxidation to MetSO is reversed by two types of Msr, A and B, specific to MetSO S- and R-diastereomers, respectively [21]. Under stress conditions, the Met residue in protein is susceptible to sulfoxide modification, which can affect protein function and is reversible by Msr^19^. Based on the Y2H screening, SlMsrB5, a potential interacting protein of SlMYC2, was obtained and further analysed. MsrB is present in most organisms and plays a critical role in responding to various stresses [22]. In pepper, CaMsrB2 improved resistance against pathogens by regulating ROS metabolism [23]. In tomato, silencing *SlMsrB2* resulted in ROS accumulation and chlorophyll degradation, inhibiting drought resistance [24]. In contrast, *SlMsrB2* overexpression improved drought resistance by decreasing ROS accumulation and causing delayed chlorophyll degradation [24]. In this study, a SelR domain was found in the C-terminus of SlMsrB5 and SlMsrB5 could result in the reduction of MetSO to Met. MeJA treatment significantly increased *SlMsrB5* expression. Moreover, Y2H, BiFC, and LCI assay further revealed that SlMsrB5 could interact with SlMYC2. And, SlMsrB5 was also involved in the repair of the oxidized methionine residue in SlMYC2. These findings indicate that SlMYC2 is the target of SlMsrB5 in tomato fruit, suggesting that SlMsrB5 is involved in MeJA–SlMYC2-mediated postharvest fruit metabolism.

In addition to transcriptional regulation, post-translational modifications of a transcription factor can affect its stability and DNA-binding ability, thus consequently regulating its regulatory effects on target genes [17]. Cyclic oxidation/reduction of Met residues in proteins, which can be reversibly regulated by Msr, is important in stress responses or disease-related biological processes [36, 37]. For example, Jiang *et al.* [25] found that the redox state of NOR regulated by MsrB2 promoted fruit ripening by increasing the transcript levels of ripening-related genes. Moreover, Yan *et al.* [26] demonstrated that MaMsrB2 participated in the redox regulation of MaNAC42 and regulated the banana ripening process. In this study, we noticed that SlMsrB5 partially repaired the binding ability of oxidized SlMYC2 to the G-box motif. The SlMYC2 protein contains eighteen methionine residues, of which Met542 is located in the bHLH domain that has been reported to be related to the DNA-binding ability [11]. Mimicking sulfoxidation of Met542 in SlMYC2 largely inhibited the transcriptional activity of SlMYC2. The results revealed a post-translational modification of SlMYC2, that is, sulfoxidation, which could regulate its DNA-binding ability and transcriptional activity. The *SlMsrB5*-silenced fruit was developed to further investigate the role of SlMsrB5 in MeJA–SlMYC2-mediated transcriptional regulation of *SlATGs*, indicating that silencing *SlMsrB5* significantly reduced *SlMYC2* and *SlATGs* expression. SlMsrB5 was found to be involved in JA biosynthesis, and silencing *SlMsrB5* significantly inhibited the JA content [28], which might inhibit *SlMYC2* expression. Furthermore, an MYC2-Dof2.1-MYC2 feedforward transcriptional loop was proposed to participate in plant metabolism [38]. As a result, we hypothesized that SlMsrB5 might be involved in the SlMYC2 redox status and transcriptional activity and that it might then regulate *SlMYC2* expression via a similar MYC2-Dof2.1-MYC2 feedforward transcriptional loop. Furthermore, DLR results indicated that the SlMsrB5–SlMYC2 interaction increased the activation effects of SlMYC2 on *SlATG13a*, *SlATG13b*, *SlATG18a,* and *SlATG18h*.

A hypothetical model is proposed to demonstrate the role of SlMsrB5 in MeJA–SlMYC2-mediated autophagic activity. SlMYC2 could regulate postharvest fruit defense responses by specifically regulating *SlATGs* expression. However, under stress conditions, ROS induced by various stresses causes methionine oxidation in SlMYC2, which inhibits its DNA-binding ability and transcriptional activity, thereby reducing *SlATGs* expression in postharvest fruit. In addition, MeJA treatment promotes the expression of *SlMsrB5*, which targets SlMYC2 for sulfoxidation, thus also maintaining the redox status of SlMYC2 and regulating the expression of *SlATGs*. These results provide new information on MeJA-mediated postharvest fruit autophagy regulation by highlighting the sulfoxidation regulation of SlMYC2.

## Materials and methods

### Fruit and treatment

*SlMYC2*-silenced and *SlMSRB5*-silenced tomato fruits (*Solanum lycopersicum* L. cv. Badun) were constructed using VIGS [15, 34]. The recombinant vectors pTRV2-*SlMYC2* and pTRV2-*SlMSRB5* were each constructed by inserting *SlMYC2* and *SlMsrB5* fragments into the vector pTRV2. Afterward, the vector pTRV1 was mixed with each of pTRV2, pTRV2-*SlMYC2,* and pTRV2-*SlMsrB5* to infect tomato fruit (10 days after pollination) with *Agrobacterium* GV3101.

The tomato fruits, including the control, *SlMYC2-*silenced, and *SlMsrB5*-silenced fruits, were collected at the green-mature stage and randomly divided into two groups. They were then treated with air and 0.05 mmol L^−1^ MeJA vapors for 12 h at room temperature in sealed containers. Following this treatment, the fruits from each treated group were stored for 12 d (25 ± 1°C, 80–90% relative humidity). Each treatment was replicated three times (50 fruits/replicate).

### Bioinformatics analysis

The domain structure of SlMYC2 was analysed using the SMART, Interpro database, and web tools of CD (Conserved Domain)-Search. The prediction of subcellular localization of SlMYC2 and SlMsrB5 was conducted using ProtCompV.9.0 Server, Target P 2.0 Server, and Cell-PLoc 2.0. Protein sequence alignment was performed by using DNAMAN BLAST.

### Gene expression analysis

Gene expression analysis was carried out using quantitative real-time PCR (qRT-PCR) on a LineGene 9600 detection system (Bioer, Hangzhou, China) with the SYBR Green I Master Mix (Toyobo, Osaka, Japan), as previously described [15]. Additionally, the primers were shown in Table S2 (see online supplementary material). The gene expression levels were figured out using the 2^-△△Ct^ method and normalized by the reference gene *SlUbi3*.

### Purification of recombinant proteins

The CDS of *SlMYC2* and *SlMsrB5* were synthesized from tomato cDNA and inserted into pET-28a, pMAL-c2X, and pCold-TF to generate pET-28a-*SlMYC2*, pMAL-c2X-*SlMYC2*, and pCold-TF-*SlMsrB5.* Recombinant vectors were transformed into *Escherichia coli* BL21 (DE3) and induced with isopropyl-1-thio-β-d-galactopyranoside (IPTG) [14]. Recombinant proteins (pET-28a-SlMYC2 and pCold-TF-SlMsrB5) were purified using Ni-NTA His-Bind® resin (GE Healthcare, Piscataway, NJ, USA), and pMAL-c2X-SlMYC2 was purified using MBPSEP Dextrin Agarose Resin 6FF (Yeasen, Shanghai, China), according to the manufacturer’s instructions. Primers for all constructs are listed in Table S3.

### EMSA assay

EMSA was performed to determine whether SlMYC2 could directly bind to *SlATGs* promoters, according to a method previously described [14]. Probes containing the G/E-box were designed and labeled with biotin. Unlabeled probes were considered as cold competitor probes. The probes with a mutated G/E-box (TTTTTTTT) were employed as mutation probes. EMSA was performed using chemiluminescent EMSA kit (Beyotime, Shanghai, China).

To assay the role of methionine sulfoxidation in SlMYC2 on its DNA-binding ability, the labeled G-box probe was mixed with each of pCold-TF-SlMsrB5, MBP-SlMYC2, oxidized MBP-SlMYC2 using H_2_O_2_, and repaired MBP-SlMYC2 using pCold-TF-SlMsrB5. The results were obtained using a chemiluminescent EMSA kit (Beyotime, Shanghai, China).

### DLR assay

The DLR assay was performed exactly as described in our previous method [14]. The CDS of *SlMYC2* and *SlMsrB5* were each sub-cloned into pGreenII-62SK to construct effector vectors pGreenII-62SK-*SlMYC2* and pGreenII-62SK-*SlMsrB5*, respectively. The promoter sequences of *SlATG13a*, *SlATG13b*, *SlATG18a,* and *SlATG18h* were each sub-cloned into pGreenII-0800-Luc to construct report vectors pGreenII-0800-Luc-*SlATG13a* pro, pGreenII-0800-Luc-*SlATG13b* pro, pGreenII-0800-Luc-*SlATG18a* pro, and pGreenII-0800-Luc-*SlATG18h* pro, respectively. All the constructs were individually transformed into *Agrobacterium tumefaciens* GV3101 (pSoup-P19). *Agrobacterium* culture mixtures including effector and reporter vectors were co-injected into tobacco leaves with needleless syringes. The values of LUC and REN were determined after 72 h using a dual-luciferase reporter gene assay kit (Beyotime, Shanghai, China). The luminescence was measured with a Chemiscope 6000 (Clinx, Shanghai, China). Primers for vector constructs are listed in Table S3.

### Y2H screening and assay

To screen the SlMYC2-interacting proteins, the CDS of *SlMYC2* was amplified using the primers shown in Table S3 and inserted into the pGBKT7 vector as bait vector pGBKT7-*SlMYC2*. The tomato cDNA library was generated, which was used to perform the Y2H screening assay. The positive clones were further determined by sequencing.

Next, the Y2H assay was used to detect the interaction of SlMYC2 and SlMsrB5, according to the method of Ji *et al.* [39]. The CDS of *SlMsrB5* was inserted into pGADT7 to construct the prey vector pGADT7-*SlMsrB5* using the primers shown in Table S3. The plasmids described above were co-transformed into the yeast strain AH109 (Weidi Biotechnology, Shanghai, China) and grown for 3 days at 29°C on an SD/−Leu/−Trp medium. Transformed colonies were incubated on an SD/−Leu/−Trp/-His/−Ade medium with and without X-α-Gal to observe the growth stage and color of the yeast colony.

### BiFC assay

The BiFC assay was conducted according to the method of Li *et al.* [40]. Recombinant vectors of SlMYC2-YNE and SlMsrB5-YCE were generated by cloning the CDS of *SlMYC2* and *SlMsrB5* into pEAQ-YNE and pEAQ-YCQ, respectively. All the constructs were individually transformed into *A. tumefaciens* GV3101. The tobacco leaves were then co-infiltrated by the *Agrobacterium* strain GV3101 harboring the above vector combinations (SlMYC2-YNE + YCE, YNE + SlMsrB5-YCE, SlMYC2-YNE + SlMsrB5-YCE). The fluorescence signals of YFP were detected using a fluorescence microscope after 3 days. Primers for all constructs are listed in Table S3.

### LCI assay

The LCI assay was carried out as described by Li *et al.* [41]. The CDS of *SlMYC2* and *SlMsrB5* were cloned with specific primers (Table S3) and inserted into pCAMBIA1300-cLuc and pCAMBIA1300-nLuc, respectively. All the constructs were individually transformed into *A. tumefaciens* GV3101. The different vector combinations (cLuc+nLuc; cLuc+nLuc-SlMsrB5; cLuc-SlMYC2 + nLuc; cLuc-SlMYC2 + nLuc-SlMsrB5) were co-injected into tobacco leaves by the *Agrobacterium* strain GV3101 in equal volumes. After 3 days, the luciferase activity and signal were measured using the Varioskan LUX Multimode Microplate Reader (Thermo) and Chemiscope 6000 (Clinx, Shanghai, China), respectively.

### Measurement of SlMsrB5 activity

The SlMsrB5 activity was measured using HPLC. The recombinant protein pCold-TF-SlMsrB5 was mixed with HEPES (pH 8.0, 15 mmol L^−1^), containing 0.03 mol L^−1^ KCl, 0.01 mol L^−1^ MgCl_2_, 0.02 mol L^−1^ DTT, and 0.5 mmol L^−1^ MetSO, for 3 h at 37°C. The above mixture, Met and MetSO were then derivatized using the method described by Zhang *et al.* [42]. The solution (20 μL) was injected into an HPLC system (Agilent 1260 Infinity II, USA) equipped with a C18 column (250× 4.6 mm, 5 μm inner diameter, Diamonsil) to separate the Met and MetSO (flow rate: 0.8 mL min^−1^; wavelength: 254 nm; column temperature: 35°C). The mobile phases consisted of 100 mmol L^−1^, pH 6.2 sodium acetate buffer (A) and acetonitrile (B). The gradient program was initiated with 20% B, increasing to 80% over 30 min.

### Oxidation and reduction assay of SlMYC2

Oxidation and reduction of SlMYC2 were measured as described by Jiang *et al.* [25]. The His-tagged SlMYC2 protein was oxidized by reacting it with 1 mmol L^−1^ H_2_O_2_ for 3 h at 37°C. Then, the mixture was centrifuged by a Millipore ultrafiltration centrifuge tube (MWCO = 30 kDa) to remove excess H_2_O_2_. The oxidized SlMYC2 was incubated with purified recombinant protein pCold-TF-SlMsrB5, containing 10 mmol L^−1^ DTT, for 3 h at 37°C. To stop the reaction, trifluoroacetic acid was added, and SDS-PAGE was performed.

Trypsin was used to digest protein bands with different redox states in gel. LC–MS/MS was performed on a Q Exactive mass spectrometer (Thermo Fisher Scientific) that was coupled to an Easy-nLC 100 (Thermo Fisher Scientific) to analyse the peptides at Applied Protein Technology Co., Ltd. (APTBIO, Shanghai, China). The relative abundance of each Met-containing peptide was determined by integrating peak area intensities.

### Mimicking of methionine sulfoxidation of SlMYC2 by site-directed mutagenesis

The effector vector pBD-*SlMYC2* was constructed using a seamless cloning kit (Beyotime, Shanghai, China). To mimic methionine sulfoxidation, Met542 in SlMYC2 was mutated to glutamine using a site-directed mutagenesis Kit (Beyotime, Shanghai, China), with the constructed vector pBD-*SlMYC2* as a template, and transformed into DH5α chemically competent cell. Using the *Agrobacterium* strain GV3101 (pSoup-P19), the effector vector and reporter vector (pGreenII 0800-LUC-TATA) were co-infected into tobacco leaves. The values of LUC and REN were determined after 72 h using a dual-luciferase reporter gene assay kit (Beyotime, Shanghai, China).

### Statistical analysis

The experiments were carried out in triplicate using a randomized design. Data analysis was performed using one-way analysis of variance and Tukey’s multiple range tests using SPSS software (version 19.0; SPSS Inc., Chicago, IL, USA). Differences were significant at *P* < 0.05. A heatmap was plotted using R software (version 3.6.1) pheatmap packages.

## Acknowledgments

This work was supported by the National Natural Science Foundation of China (No. 32172278) and the Shandong Province Natural Science Foundation (ZR2020KC011). We are grateful to City Hills Proofreading for their assistance in editing this manuscript.

## Author contributions

D.M., F.L., and X.Z. conceived and designed experiments; D.M., J.L., and X.F. conducted the experiment; D.M., Y.S., J.D., and X.L analysed the data; D.M. and F.L. wrote the manuscript; X.Z. and F.L. revised the manuscript. N.J. provided technical support. M.A. improved the language. X.Z. supervised the experiment. All authors read and approved the manuscript.

## Data availability

All data generated and analysed during this study are included in this article (and supplementary file).

## Conflicts of interest statement

The authors declare no conflict of interest.

## Supplementary Data

Supplementary data is available at *Horticulture Research* online.

## Supplementary Material

Web_Material_uhad012
