# Supplementary material for: Interaction of methionine sulfoxide reductase B5 with SlMYC2 stimulates the transcription of MeJA-mediated autophagy-related genes in tomato fruit
Source: Hortic Res. 2023 Feb 1;10(3):uhad012. doi: 10.1093/hr/uhad012 (PMC10031729; doi:10.1093/hr/uhad012)
Supplement: Web_Material_uhad012 [file web_material_uhad012.zip › SUPPLEMENTARY INFORMATION.doc]

**SUPPLEMENTARY INFORMATION**

**Interaction of methionine sulfoxide reductase B5 with SlMYC2 stimulates the transcription of MeJA-mediated autophagy-related genes in tomato fruit**

Dedong Min^1,#^, Fujun Li^1,#^, Maratab Ali^1,2^, Jiong Liu^1^, Xiaodong Fu^1^, Yanan Song^1^, Jun Ding^1^, Xiaoan Li^1^, Nana Ji^1^, Xinhua Zhang^1,^*

^1^ School of Agricultural Engineering and Food Science, Shandong University of Technology, Zibo 255000, Shandong, PR China

^2^ School of Food and Agricultural Sciences, University of Management and Technology, Lahore 54000, Pakistan.

Correspondence: Xinhua Zhang (E-mail: [zxh@sdut.edu.cn](mailto:zxh@sdut.edu.cn), Tel/Fax: +86-533-2786398)

^#^ These authors contributed equally.

**Table S1 The effect of *SlMYC2* silence on MeJA-mediated transcript levels of autophagy-related genes in tomato fruit during postharvest storage periods.**

|  | Treatment |  | | Storage (h) | | | | |
| --- | --- | --- | --- | --- | --- | --- | --- | --- |
|  |  | 0 | 3 | | 6 | 9 | 12 | |
| *SlATG2* | Control | 1.00±0.00a | 1.57±0.21c | | 4.83±0.62a | 4.63±0.29b | 3.38±0.35b |  |
|  | MeJA | 1.00±0.00a | 3.87±0.46a | | 4.12±0.46a | 9.96±1.13a | 4.43±0.29a |  |
|  | *SlMYC2*-silenced | 0.81±0.09b | 1.06±0.07d | | 2.78±0.06b | 2.94±0.18c | 1.98±0.16d |  |
|  | *SlMYC2*-silenced+MeJA | 0.81±0.09b | 2.13±0.38b | | 3.93±0.32a | 4.63±0.16b | 2.72±0.15c |  |
| *SlATG5* | Control | 1.00±0.00a | 4.82±0.22b | | 4.11±0.12b | 6.08±0.24b | 3.37±0.44b |  |
|  | MeJA | 1.00±0.00a | 12.36±1.20a | | 5.94±0.18a | 7.82±0.31a | 8.70±0.22a |  |
|  | *SlMYC2*-silenced | 0.58±0.09b | 2.62±0.25c | | 3.15±0.12c | 3.44±0.40d | 1.51±0.24c |  |
|  | *SlMYC2*-silenced+MeJA | 0.58±0.09b | 4.31±0.42b | | 4.29±0.42b | 5.28±0.39c | 3.70±0.32b |  |
| *SlATG6* | Control | 1.00±0.00a | 1.62±0.14c | | 3.15±0.24a | 1.60±0.17b | 2.26±0.32b |  |
|  | MeJA | 1.00±0.00a | 3.39±0.33a | | 2.66±0.28a | 2.74±0.45a | 3.45±0.27a |  |
|  | *SlMYC2*-silenced | 0.26±0.03b | 0.91±0.15d | | 1.02±0.17c | 1.23±0.23c | 1.15±0.18c |  |
|  | *SlMYC2*-silenced+MeJA | 0.26±0.03b | 2.34±0.45b | | 1.89±0.23b | 1.82±0.31b | 1.80±0.23b |  |
| *SlATG7* | Control | 1.00±0.00a | 1.96±0.22b | | 3.31±0.17a | 1.95±0.23b | 1.48±0.08c |  |
|  | MeJA | 1.00±0.00a | 6.14±0.85a | | 3.44±0.25a | 4.39±0.62a | 3.08±0.07a |  |
|  | *SlMYC2*-silenced | 0.20±0.02b | 0.74±0.20c | | 1.91±0.04c | 0.92±0.02c | 1.21±0.13d |  |
|  | *SlMYC2*-silenced+MeJA | 0.20±0.02b | 2.29±0.35b | | 2.66±0.43b | 1.97±0.12b | 1.89±0.19b |  |
| *SlATG8a* | Control | 1.00±0.00a | 1.58±0.19a | | 1.03±0.06c | 0.81±0.11c | 0.93±0.14c |  |
|  | MeJA | 1.00±0.00a | 1.87±0.18a | | 6.31±0.33a | 3.27±0.35a | 5.11±0.27a |  |
|  | *SlMYC2*-silenced | 0.49±0.02b | 0.89±0.13b | | 0.90±0.07c | 0.59±0.04d | 0.55±0.05d |  |
|  | *SlMYC2*-silenced+MeJA | 0.49±0.02b | 1.08±0.09b | | 2.48±0.30b | 1.29±0.15b | 1.97±0.29b |  |
| *SlATG8c* | Control | 1.00±0.00a | 2.32±0.26b | | 2.35±0.20b | 1.56±0.08b | 0.75±0.05c |  |
|  | MeJA | 1.00±0.00a | 3.79±0.34a | | 4.41±0.16a | 2.65±0.23a | 2.51±0.39a |  |
|  | *SlMYC2*-silenced | 0.60±0.05b | 0.92±0.16d | | 0.82±0.11d | 1.18±0.12c | 0.66±0.09c |  |
|  | *SlMYC2*-silenced+MeJA | 0.60±0.05b | 1.43±0.14c | | 1.88±0.16c | 1.45±0.11b | 1.81±0.11b |  |
| *SlATG8f* | Control | 1.00±0.00a | 0.94±0.15b | | 1.19±0.19b | 0.81±0.14c | 1.12±0.13b |  |
|  | MeJA | 1.00±0.00a | 1.94±0.19a | | 1.79±0.01a | 1.94±0.19a | 1.70±0.23a |  |
|  | *SlMYC2*-silenced | 0.51±0.09b | 0.47±0.07c | | 1.02±0.15b | 0.78±0.09c | 0.56±0.06c |  |
|  | *SlMYC2*-silenced+MeJA | 0.51±0.09b | 1.14±0.10b | | 0.98±0.04b | 1.23±0.11b | 1.40±0.07b |  |
| *SlATG8h* | Control | 1.00±0.00a | 0.48±0.06b | | 0.84±0.11b | 0.55±0.07b | 0.43±0.05b |  |
|  | MeJA | 1.00±0.00a | 0.98±0.06a | | 0.96±0.12a | 1.10±0.09a | 0.81±0.13a |  |
|  | *SlMYC2*-silenced | 0.33±0.02b | 0.65±0.11b | | 0.40±0.09c | 0.41±0.06c | 0.38±0.06b |  |
|  | *SlMYC2*-silenced+MeJA | 0.33±0.02b | 0.60±0.11b | | 0.51±0.06c | 0.67±0.09b | 0.72±0.05a |  |
| *SlATG10* | Control | 1.00±0.00a | 0.62±0.09b | | 1.49±0.21a | 0.37±0.05b | 0.67±0.05c |  |
|  | MeJA | 1.00±0.00a | 1.93±0.24a | | 1.37±0.02a | 0.95±0.15a | 1.36±0.22b |  |
|  | *SlMYC2*-silenced | 0.42±0.05b | 0.45±0.02c | | 0.34±0.05c | 0.34±0.02b | 0.35±0.03d |  |
|  | *SlMYC2*-silenced+MeJA | 0.42±0.05b | 0.69±0.02b | | 1.12±0.15b | 0.86±0.04a | 2.06±0.23a |  |
| *SlATG13a* | Control | 1.00±0.00a | 1.49±0.22c | | 2.08±0.26c | 4.77±0.18b | 4.80±0.31b |  |
|  | MeJA | 1.00±0.00a | 4.36±0.37a | | 6.41±0.58a | 12.81±0.84a | 3.51±0.13c |  |
|  | *SlMYC2*-silenced | 0.36±0.03b | 0.83±0.10d | | 1.05±0.01d | 1.26±0.27c | 1.21±0.04d |  |
|  | *SlMYC2*-silenced+MeJA | 0.36±0.03b | 3.80±0.47b | | 3.83±0.19b | 4.44±0.20b | 6.22±0.87a |  |
| *SlATG13b* | Control | 1.00±0.00a | 0.86±0.07b | | 0.99±0.12b | 1.23±0.05b | 0.61±0.06b |  |
|  | MeJA | 1.00±0.00a | 1.48±0.20a | | 1.69±0.17a | 2.59±0.28a | 1.25±0.03a |  |
|  | *SlMYC2*-silenced | 0.49±0.08b | 0.53±0.06c | | 0.46±0.03c | 0.36±0.05c | 0.53±0.08b |  |
|  | *SlMYC2*-silenced+MeJA | 0.49±0.08b | 0.90±0.10b | | 1.03±0.07b | 1.54±0.13b | 1.12±0.12a |  |
| *SlATG18a* | Control | 1.00±0.00a | 0.89±0.05b | | 1.02±0.08b | 1.21±0.10b | 0.89±0.02c |  |
|  | MeJA | 1.00±0.00a | 1.11±0.20a | | 1.20±0.03a | 2.16±0.29a | 1.41±0.09b |  |
|  | *SlMYC2*-silenced | 0.15±0.01b | 0.68±0.10c | | 0.54±0.05c | 0.49±0.01c | 0.28±0.03d |  |
|  | *SlMYC2*-silenced+MeJA | 0.15±0.01b | 0.59±0.08c | | 1.04±0.15b | 1.29±0.16b | 1.79±0.04a |  |
| *SlATG18f* | Control | 1.00±0.00a | 0.78±0.06b | | 1.99±0.14b | 1.81±0.26b | 0.51±0.02c |  |
|  | MeJA | 1.00±0.00a | 2.30±0.20a | | 2.76±0.13a | 4.54±0.31a | 3.86±0.34a |  |
|  | *SlMYC2*-silenced | 0.51±0.04b | 0.95±0.13b | | 1.10±0.16c | 1.16±0.08c | 0.30±0.03d |  |
|  | *SlMYC2*-silenced+MeJA | 0.51±0.04b | 0.82±0.07b | | 2.70±0.27a | 1.87±0.17b | 1.25±0.16b |  |
| *SlATG18h* | Control | 1.00±0.00a | 0.49±0.04c | | 0.74±0.08b | 1.10±0.02b | 1.00±0.14b |  |
|  | MeJA | 1.00±0.00a | 1.57±0.13a | | 1.48±0.15a | 2.11±0.23a | 1.85±0.23a |  |
|  | *SlMYC2*-silenced | 0.22±0.03b | 0.30±0.04d | | 0.53±0.04c | 0.66±0.01c | 0.42±0.03c |  |
|  | *SlMYC2*-silenced+MeJA | 0.22±0.03b | 0.68±0.13b | | 0.76±0.09b | 1.02±0.14b | 0.96±0.07b |  |

Data are expressed as the means±standard errors. Means in a column followed by a different letter for each gene differ significantly at p < 0.05 by Tukey’s multiple range tests.

**Table S2 List of primers used in quantitative real-time PCR.**

| Genes | Accession numbers | Primers（5’-3’） |
| --- | --- | --- |
| *SlATG2* | Solyc01g108160 | Forward: GTCATTGAAGAGGCACTGCT  Reverse: AGCTAAATCAACACGGCAAG |
| *SlATG5* | Solyc02g036380 | Forward: TCAGATGGTGCTGAGATCAAG  Reverse: ATTGTTTACCACCCATGCA |
| *SlATG6* | Solyc05g050390 | Forward: CCCATGCAGTCAAACAATTC  Reverse: CCCTCATGCATTCAAGAC |
| *SlATG7* | Solyc11g068930 | Forward: ATTCAACGGCTAACCGTACC  Reverse: CAAACTCAGCTTTGGCACAT |
| *SlATG8a* | Solyc07g064680 | Forward: ACCGGTGATTGTTGAGAAGG  Reverse: GCGCTGAGCTTAATCCTCTT |
| *SlATG8c* | Solyc03g031650 | Forward: TTGGCCAATTTGTTTACGTT  Reverse: AAAGGAATCCGTCTTCATCC |
| *SlATG8f* | Solyc08g078820 | Forward: GGCAATCATGTCTGCAATCT  Reverse: TCAAAGCTACAGTTCGCTCAG |
| *SlATG8h* | Solyc01g068060 | Forward: CGTGTTTGTGAATAACACCTTG  Reverse: AGCACATGTAGAGGAACCCA |
| *SlATG10* | Solyc09g047840 | Forward: GGAGAACCCTTGGCAATAGA  Reverse: TAGTCCCACATGGATGCAAT |
| *SlATG13a* | Solyc03g096790 | Forward: GATGTCGACACTTCCGATTC  Reverse: TTGCAGATCCCTGAGAAGAG |
| *SlATG13b* | Solyc06g072980 | Forward: CTGTAGGTGCCCTTGTTCAC  Reverse: AAGCTTTGAGCTCCTCCAAT |
| *SlATG18a* | Solyc08g006010 | Forward: CAGCGAGTTCACCACTATCC  Reverse: TCCATCCAAGCCAAGAATTA |
| *SlATG18f* | Solyc12g005230 | Forward: TCCGAAGCAGAACTCCAAAT  Reverse: AACCTCAGCCTCTCCACGAC |
| *SlATG18h* | Solyc07g064060 | Forward: GGCTAAATACAGTGAGCAATG  Reverse: GTGAAAATCAGCAGCAAGAAC |
| *SlMYC2* | Solyc08g076930 | Forward: CAGTTTTGCCTTCTTCGGGC-3  Reverse: TTCGCTGGCTTTCTACCTCG- |

**Table S3** List of primers used in this study.

| Assay | Genes | Accession numbers | Primers（5’-3’） |
| --- | --- | --- | --- |
| pET-28a/ pMAL-c2X DLR/Y2H | *SlMYC2* | Solyc08g076930 | Forward: ATGGATCCATGACTGAATACAGCTTGCCCACC  Reverse:GCGTCGACCTAGTGTGTTTCAGCAATTTTCGATGTCAAC |
| pCold-TF | *SlMsrB5* | Solyc10g047930 | Forward: cgGGATCCatgggttctcatattctcaaaatctcac  Reverse: GCGTCGACctaagaatttgctggtgtaaacttgag |
| DLR | *SlATG13a* | Solyc03g096790 | Forward:CGCGTCGACCAAATATATCTTCTCATTAGATGAAAAATC  Reverse: CGGGATCCCATTTAATATACCCCAAACAAAAATC |
| DLR | *SlATG13b* | Solyc06g072980 | Forward: CGCGTCGAC ATTTTGCTGGCATTATCGTCA  Reverse: CGGGATCC CTTTTTTGTCTTTCAAACAGATGC |
| DLR | *SlATG18a* | Solyc08g006010 | Forward: CGCGTCGAC GGTTCTGCAATAAATTTTTATACG  Reverse: CGGGATCC TGGACGGATTAGGAAAAAATC |
| DLR | *SlATG18h* | Solyc07g064060 | Forward: CGCGTCGACGTAATAAAGAATATGTGCTCCTCGCTC  Reverse: CGGGATCCTCACCGGTAACCAGCAAGT |
| DLR | *SlMsrB5* | Solyc10g047930 | Forward: GCTCTAGAatgggttctcatattctcaaaatctcac  Reverse: GCGTCGACctaagaatttgctggtgtaaacttgag |
| Y2H | *SlMsrB5* | Solyc10g047930 | Forward: CGGAATTCAtgggttctcatattctcaaaatctcac  Reverse: CGGGATCCctaagaatttgctggtgtaaacttgag |
| BiFC | *SlMYC2* | Solyc08g076930 | Forward: ATATTCTGCCCAAATTCGCGatgactgaatacagcttgcccacc  Reverse: CCCTTGCTCACCATACCGGTCGCgtgtgtttcagcaattttcgatg |
| BiFC | *SlMsrB5* | Solyc10g047930 | Forward: ATATTCTGCCCAAATTCGCGatgggttctcatattctcaaaatctcacc  Reverse: TGCACGCTGCCACCGGTCGCagaatttgctggtgtaaacttgagg |
| LCI | *SlMYC2* | Solyc08g076930 | Forward:GGGGTACCATGACTGAATACAGCTTGCCCACC  Reverse:CGGGATCCTTAGTGTGTTTCAGCAATTTTCGATGTCAAC |
| LCI | *SlMsrB5* | Solyc10g047930 | Forward: CGGGATCCatgggttctcatattctcaaaatctcac  Reverse: GCGTCGACctaagaatttgctggtgtaaacttgag |
| VIGS | *SlMsrB5* | Solyc10g047930 | Forward:CGGAATTCTCATCCCAAGACCCAATTCAGGTTT  Reverse: CGGGATCCTCTCAACCCTCCTGCCATCAG |

Figure S1. The analysis of site-directed mutagenesis in *SlMYC2*.
